# Hispanic ancestry as a modifier of neuroinflammatory gene expression in single and mixed etiology dementias

**DOI:** 10.1002/alz70855_106709

**Published:** 2025-12-24

**Authors:** Elizabeth Ochoa, Savannah Barannikov, Mallory Keating, Margaret E Flanagan, Brittany N Dugger, Dennis W. Dickson, Kevin F. Bieniek

**Affiliations:** ^1^ Glenn Biggs Institute for Alzheimer's and Neurodegenerative Diseases, UT Health San Antonio, San Antonio, TX, USA; ^2^ Glenn Biggs Institute for Alzheimer's & Neurodegenerative Diseases, University of Texas Health Sciences Center at San Antonio, San Antonio, TX, USA; ^3^ University of California, Davis, Sacramento, CA, USA; ^4^ Mayo Clinic, Jacksonville, FL, USA

## Abstract

**Background:**

Hispanic individuals are at a 1.5‐times higher risk for Alzheimer's disease and related dementias yet remain underrepresented in research. Longitudinal Hispanic cohorts provide antemortem biospecimen, cognitive, and epidemiological data, and GWAS/admixture mapping analyses identify Hispanic ancestry as a modifier of Alzheimer's disease‐associated gene expression. Additionally, antemortem‐based studies suggest an altered pathological burden in Hispanic individuals, with neuropathological reports revealing a disproportional anatomy‐dependent burden of Alzheimer's pathologies in Hispanic decedents. Despite these advances, few analyses investigate differential gene expression in dementia from postmortem brain of Hispanic decedents. To address the current knowledge gap, we investigated neuroinflammatory gene expression in Hispanic decedents with single or mixed etiology dementia.

**Method:**

To quantify gene expression, RNA was extracted from postmortem frontal cortex brain tissue of Hispanic and non‐Hispanic decedents with Alzheimer's disease (AD, *n* = 6), cerebrovascular disease (CVD, *n* = 6), or a mixture of the two etiologies (ADCVD, *n* = 6). Following quality control, extracted RNA was hybridized to the NanoString Human Neuroinflammation panel, and gene expression quantified via SPRINT nCounter. Analysis was completed with NanoString Advanced Analysis criteria via ROSALIND®. Low counts and low background genes were omitted from analysis.

**Result:**

Comparison of Hispanic decedents with either AD or ADCVD to non‐Hispanic decedents reveals shared pathway enrichment of the Astrocyte Function gene set. Respective to non‐Hispanic decedents, we find two genes in Hispanic AD and seven genes in Hispanic ADCVD that are differentially expressed. Across all samples, gene expression analysis indicates *P2RY12, NRP2, PRKACB*, and seven other genes are increased at the transcript level in the frontal cortex of Hispanic decedents with CVD when compared to either Hispanic or non‐Hispanic decedents with other etiologies.

**Conclusion:**

These preliminary findings suggest an ancestry‐ and etiology‐specific transcriptional profile in CVD, and point to few genes modified by ancestry in AD and ADCVD. Ongoing experiments aim to determine differential gene expression relative to pathology, and to develop functional genetic models for screening. Overall, we seek to identify Hispanic ancestry‐ and disease‐specific genes among dementia etiologies as an avenue for precision medicine dementia care among an at‐risk, underrepresented population.

